# Goal-Conflict EEG Theta and Biased Economic Decisions: A Role for a Second Negative Motivation System

**DOI:** 10.3389/fnins.2020.00342

**Published:** 2020-04-15

**Authors:** Phoebe S.-H. Neo, Jessica Tinker, Neil McNaughton

**Affiliations:** Department of Psychology, University of Otago, Dunedin, New Zealand

**Keywords:** theta, uncertainty, decision bias, conflict, anxiety, emotion, approach-avoidance, RST

## Abstract

Economic decision biases can reflect emotion and emotion dysfunction. Economic paradigms thus provide a solid framework for analysis of brain processes related to emotion and its disorders. Importantly for economic decisions, goal-conflict activates different negative motivational processes than pure loss; generating negative decision biases linked to anxiety and fear, respectively. Previously, right frontal goal-conflict specific EEG rhythmicity (GCSR) was shown to reflect anxiety processing. Here, we assessed GCSR in a forced-choice, economic decision-making task. Ninety participants were tested in three key conditions where gain:loss ratios of left mouse clicks were set to 75:25 (GAIN), 50:50 (CONFLICT) and 25:75 (LOSS). Right clicks produced no monetary consequences and skipped the current trial. The participants were not told the different conditions but could learn about them by associating the background stimulus color with the specific payoff. Goal-conflict was defined as the mathematical contrast of activity in CONFLICT minus the average of that in GAIN and LOSS. Replicating previous findings with somewhat different conditions, right frontal GCSR was detected. Importantly, greater right frontal GCSR significantly predicted a preference for economic safety in CONFLICT but not in GAIN or LOSS; but did not predict trait anxiety or neuroticism. We conclude that goal-conflict has unique neuroeconomics effects on choice biases; and that these reflect anxiety processing that is not effectively captured by trait anxiety or neuroticism.

## Introduction

The study of human decision-making in economics provides clear examples of decision biases. Classic examples include over-representing sure and rare events (Kahneman and Tversky, 2013), ambiguity aversion in the Ellsberg paradox (Ellsberg, 1961), loss aversion in Prospect theory (Kahneman, 1979), and “framing effects” (Tversky and Kahneman, 1981). The field has also developed tight definitions of the factors (Glimcher and Fehr, 2013) that affect economic decisions, such as the assessments of context (history of consequences in reinforcement learning), unknowns (availability of information), chance occurrences (probability), and valuation (sensitivities to gains and losses).

Importantly, emotion affects economic choices. Hence, using economic paradigms to study the neural processes in emotional dysfunction and the development of *psychiatric* disorders, provides instant access to existing detailed neuroeconomics analysis (Kishida et al., 2010; Hasler, 2012; Sharp et al., 2012). Particularly, the economic paradigms allow us to test for extreme sensitivities, not only to gain and loss but, also goal conflict (when the possibility of both gain and loss generates approach-avoidance conflict). This links back to existing neuropsychological analysis, which suggests links between: (a) low punishment sensitivity and psychopathy (Corr and McNaughton, 2014); (b) high punishment sensitivity to fear and phobic disorders (McNaughton, 2011, 2019); low conflict sensitivity to ADHD-inattentive (Sadeghi et al., 2019); and of specific relevance here, high conflict sensitivity to anxiety (Gray and McNaughton, 2000).

Current neurally detailed theories of anxiety are based on the study of threat in rodents and generalization to humans. A hierarchy of defensive behaviors in rodents (Blanchard and Blanchard, 1990) and humans (Blanchard, 2017) matches a hierarchical neural organization based on rodent work (Gray and McNaughton, 2000; McNaughton and Corr, 2004) that also appears to apply to humans (McNaughton, 2019). This map distinguishes a Fight-Flight-Freeze System (FFFS) from a Behavioral Inhibition System (BIS). The FFFS mediates processes relating to fear of danger/unmixed threat (loss); and the BIS mediates processes relating to anxiety/goal conflict/mixed threat (loss + gain) – when appetitive and aversive goals are balanced (Gray and McNaughton, 2000). Separating the FFFS from the BIS behaviorally in human decision-making is challenging, since the BIS amplifies already existing negative behavioral tendencies *concurrently* mediated by the FFFS.

Human brain activity has been linked to the processing of anxiety in a Stop-Signal Task (SST) (Verbruggen and Logan, 2009), which does not have any explicit payoffs. Specifically, electroencephalographic (EEG) rhythmicity (4–12 Hz, i.e., spanning the conventional theta and alpha bands) was detected in the right frontal scalp area F8 (Neo et al., 2011; McNaughton et al., 2013; Shadli et al., 2016) in an analog of approach-avoidance (goal) conflict. In the SST, participants make a mouse click as fast as they can in response to a go-cue. On some trials, a stop-signal occurs unpredictably at variable delays after the go-cue (producing easy, intermediate, and difficult stopping), and participants have to withhold clicking the mouse. Goal-conflict was presumed to occur more in intermediate stop-signal delays (generating 50% successful inhibition of the mouse click with stopping and going tendencies roughly equal) and so could be extracted by contrasting intermediate against short and long delays.

Importantly, this right frontal goal-conflict-specific rhythmicity (GCSR) in the SST is sensitive to all classes of anxiolytic drug, and can be considered a biomarker of a process specific to anxiety (McNaughton et al., 2013; Shadli et al., 2015b; McNaughton, 2018). These studies also showed modest correlations of GCSR with trait anxiety (Spielberger et al., 1983), or neuroticism (Eysenck and Eysenck, 1994) – personality traits associated with anxiety (Bishop and Forster, 2013). Consistent with other studies on the SST, the conflict response appears to be localized to the right inferior frontal gyrus (Shadli et al., 2015a). However, across the SST studies, GCSR consistently did not predict stop inhibition reaction times, a standard measure of overt behavior in the SST.

Neo and McNaughton (2011) tested for the effects of goal-conflict in an economic context using a forced choice decision-making task. In their key condition (CONFLICT), potential gains and losses had values that were *known* (+10/−10 cents) but were equivocal (50:50 probability). Specificity to goal-conflict was achieved by a mathematical contrast of CONFLICT with net gain (GAIN) and net loss (LOSS), with a fourth background pure gain condition excluded from analysis. In theory, CONFLICT would concurrently activate roughly equal but incompatible goals/behavioral tendencies (McNaughton, 2011; McNaughton et al., 2016) and induce specific behaviors (e.g., risk assessment, inhibition of ongoing pre-potent responses, passive avoidance) to resolve the goal-conflict. In GAIN and LOSS, approach and avoidance tendencies would dominate, respectively. Critically, for many processes such as net payoff value, the average of GAIN and LOSS should be equivalent to CONFLICT. However, the process of goal conflict should be maximal in CONFLICT and so directly estimated by the subtraction of the GAIN + LOSS average from CONFLICT. As with the SST, the Neo and McNaughton (2011) economic task generated GCSR.

However, unlike the SST, the GCSR generated by this economic conflict did not correlate with trait anxiety or neuroticism. More importantly, like the SST, a link with overt behavior, i.e., economic decisions, was not observed.

Anxiety is hyper-sensitive to uncertainty (Grupe and Nitschke, 2013; Carleton, 2016; Tanovic et al., 2018). But, when we consider its effects on economic choice, we must note that neuroeconomics distinguishes two forms of uncertainty: risk and ambiguity. These are defined as contexts in which probabilities about economic outcomes are known and unknown, respectively (Bach and Dolan, 2012). Consistent with this, previous studies (Zhang et al., 2015; Smith et al., 2016) showed that trait anxiety and decision-making were related only under ambiguity and not under risk. Hence, the lack of correlations in the Neo and McNaughton (2011) study could be due to the fact that decision-making was made under risk not ambiguity.

So, in the current study, we set up decision-making under ambiguity by removing the information about outcome values from the Neo and McNaughton (2011) task. Not only did net value in a condition have to be learned but net value was controlled via probability with fixed payoffs rather than varying payoffs at fixed probability (50:50). We predicted that under these conditions, goal-conflict rhythmicity in the right frontal scalp site F8 would be correlated with decision-making, and with trait anxiety and neuroticism.

## Materials and Methods

### Participants

Ninety participants were recruited from the University of Otago Student Job Search. They took part in variants of the economic decision-making paradigm in Neo and McNaughton (2011), referred to here as “LEARN” (15 females and 14 males) and “TRIM” (29 females and 27 males), respectively. Ages ranged from 19–25 years. Participants were compensated with cash at hourly rates slightly above the minimum wage rate at the time of testing. All participants identified themselves as right-handers and did not report any psychological treatment in the past year. Ethical approval was provided by the Lower South Regional Committee and the University of Otago Human Ethics Committee (OTA/04/03/019).

### Data Acquisition

Electro-caps (Electro Cap International, United States) mounted with pure tin electrodes were used for recordings. Three caps, large (580–620 mm), medium (540–580 mm) and small (500–540 mm) were used to accommodate different head circumferences. EEG data were recorded from F7, F3, Fz, F4, F8, T3, C3, Cz, C4, T4, T5, P3, Pz, P4, T6. EEG was also recorded from Fp1, to detect the occurrence of eye blinks. The electrodes were referenced to activity averaged across the two earlobes, recorded with clip-on pure tin ear electrodes. Electrodes on the caps were filled with Electro Cap International Electro-Gel. Impedances were checked with a General Devices impedance meter (EIM 107-37A, United States). Mindset Model MS-1000 hardware (Nolan Computer Systems, United States) was used to capture, amplify and digitize the EEG signals at a 128 Hz sample rate with 1.8–36 Hz bandpass filters. EEG recording software controlling the MindSet was written in Visual Basic and formed part of the same program that controlled the experiments.

### Questionnaires

Questionnaires included measures of neuroticism and extraversion in the Eysenck Personality Questionnaire-Revised (EPQ-R) (Hodder and Stoughton, United Kingdom), and the Spielberger State-Trait Anxiety Inventory (STAI) (Mind Garden Inc., Menlo Park, CA, United States).

### Task Stimuli

**FIGURE 1:**
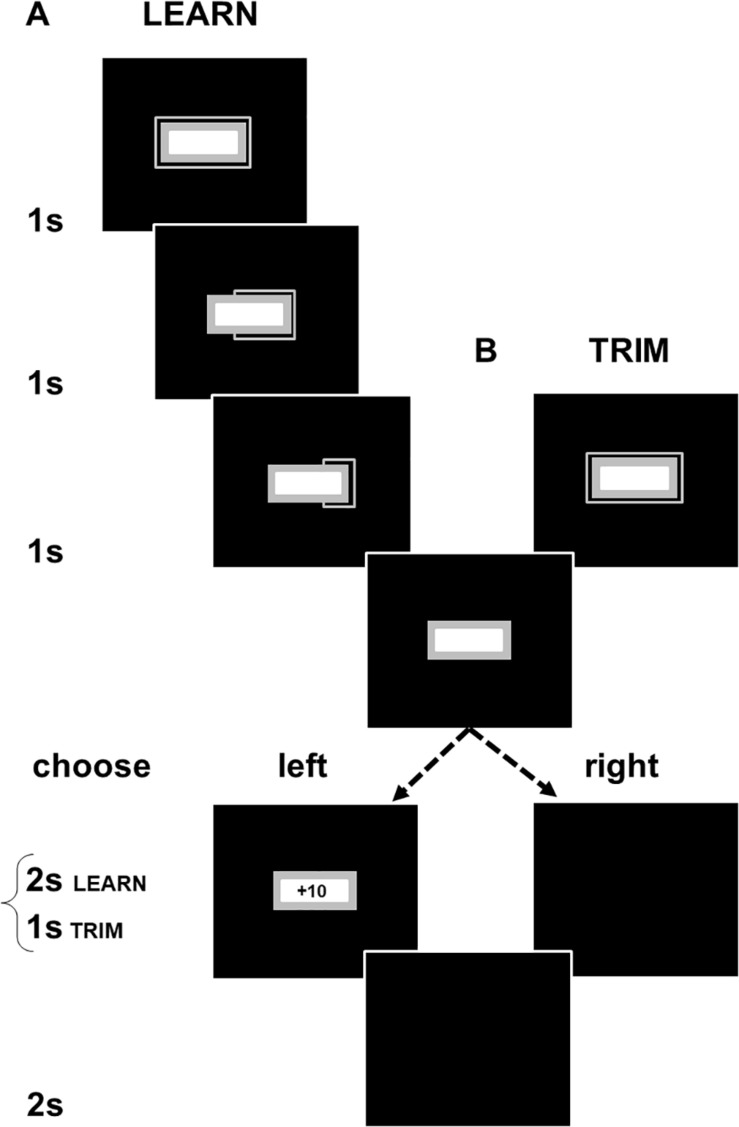
Schematic illustrations of **(A)** LEARN and **(B)** TRIM. Participants had to choose to left or right click at the end of a 3000 ms countdown period (in TRIM, the countdown and feedback periods were shortened to 1 s). Left clicks produced either a gain or loss of 10 cents. Right clicks produced no monetary consequences and allowed the participant to proceed to the next trial. Gain:loss ratios were adjusted across three color-cued experimental conditions. Participants were not informed of this but, over successive trials, could learn the association of the color cues with the probabilistic payoffs in each condition (gray box in Figure 1 showed a different color in each condition). The interval between a click and the start of the next trial was the same with both left and right clicks even though a left click produced feedback and a right click did not. There was, thus, no time incentive for participants to make either a left or right click. Participants were not given details of the timing of the task components and were only informed that the computer task would take about 45 min (TRIM: 20 min).

Here, we describe the methods for LEARN. TRIM was set up as a shorter task to address experimental fatigue in LEARN (see exclusion of participants due to artifacts in EEG processing). The changes in TRIM are indicated in brackets below. The computer stimuli in each part of the sequence of events in an experimental trial are shown in Figure 1. A trial starts with the presentation of a colored rectangular box with a frame on the outside and a smaller white box on the inside. The frame shrunk every 1,000 ms until it disappeared after 3,000 ms (TRIM: 1,000 ms). A choice of a left or right mouse click was required to call up the next stimuli. A left click produced a gain or loss with a fixed absolute value of +10/−10 cents. Gain:loss ratios of left clicks varied with experimental conditions and were set to 75:25 (GAIN), 50:50 (CONFLICT) and 25:75 (LOSS). A right click displayed a blank screen with no monetary consequences. The feedback for both left and right clicks lasted for 2 s (TRIM: 1,000 ms). The inter-trial interval consisted of a blank screen presented for 2 s.

The larger rectangular box and its outer frame (gray areas in Figure 1) displayed different colors depending on the experimental conditions. GAIN was in aquamarine [RGB (0, 255, 255)], CONFLICT in brown [RGB (139, 69, 19)], and LOSS in purple [RGB (72, 61, 139)]. LEARN included a fourth Continuous Gain condition in green [RGB (0, 100, 0)]. Practice trials were in gray [RGB (169, 169, 169)]. The stimuli were presented against a blue background (RGB [0, 0, 255], black areas in Figure 1). 10 practice trials and eight, 10-trial, blocks from each payoff condition with optional rest breaks between trial-blocks were presented. The order of the payoff conditions across and within trial-blocks was counter-balanced. The sequences of the payoffs were fixed across participants so that right clicks did not alter the pre-determined consequences of the next left click, i.e., the sequence of payoffs experienced by a participant in each of the payoff conditions was the same for all participants, regardless of when right clicks were made.

### Procedures

Participants filled out consent forms and the questionnaires upon arrival, followed by EEG preparation and the experimental task. They were instructed to make as much money as possible. On top of the compensation they received for participating in the experiment, participants had a chance to earn a bonus. In LEARN, they received a bonus amount made above $9.50. In TRIM, they were given the actual amount made during the task. There was no penalty if earnings were in deficit by the end of the experiment. The experimenter used the practice trials to demonstrate the general consequences of a left and right click, but did not inform the participants that there were different payoff conditions that were color coded. Hence, participants had to learn the payoff condition via the consistent relation of each stimulus color to a particular payoff. Participants were informed of the amount of their earnings after they completed the task. After clean up, they received payment for their participation, together with bonus earnings from the task, if any.

### EEG Processing

Ocular artifacts in the EEG were removed automatically by fitting a template to the ballistic components of eye blinks recorded on Fp1 (Zhang et al., 2017) and then removal of the fitted components from each channel scaled via linear regression (Gratton, 1998). Remaining artifacts were removed manually by deletion and were replaced with missing data markers. Deletions were always made across all channels for the relevant time period.

After artifact removal, we extracted Fast Fourier Transforms (FFT) for nominal 0.5 s epochs. A 1 s overlapping Hanning window was centered on the midpoint of the 0.5 s period of interest with 0.25 s leading and trailing overlaps. If any datum in an epoch was a missing value, the entire FFT was set to missing values. The data were then log transformed to normalize error variance, and then averaged across trials for the same payoff condition. If more than 30% of the trials contributing to the averaged power spectrum for a participant contained missing data for the same time period, the averaged spectrum for that period was replaced with missing data markers. Participants were excluded if missing values for the segments to be analyzed exceeded 10%. 11 participants showed a large number of movement EEG artifacts toward the end of LEARN, probably due to fatigue. We therefore tested more participants with a shortened version of the task, TRIM. No participants had to be excluded in TRIM due to artifacts.

### Statistical Analyses

All statistical analyses were conducted with Analysis of Variance (ANOVA) and linear regression in SPSS. Where appropriate, we extracted linear (lin) and quadratic (quad) trends using orthogonal polynomial contrasts.

#### Behavioral Analyses

We examined the effects of payoff conditions (**P**), gender (**G**), trial-blocks (**B**) and Choice task (**C**) on the number of left clicks made in each 10-trials block.

#### EEG Analyses

Consistent with Neo and McNaughton (2011), conflict and loss-gain activity were separately analyzed in the theta and alpha bands; and in the early and late phases of the tasks, respectively. Power spectra in the theta and alpha bands were averaged across 4–7 Hz and 9–12 Hz; and trials in the early and late phases were averaged across the first and last 30 trials. Conflict activity was examined with quadratic contrasts of payoff conditions (**P**). Mathematically, this is equivalent to subtracting power averaged across GAIN and LOSS from CONFLICT. Loss-gain activity was analyzed with the linear contrasts. Mathematically, this is the equivalent of subtracting power in GAIN from LOSS, while ignoring CONFLICT. Linear and quadratic contrasts were extracted for “Site” (**S**) with F7, F3, Fz, F4, and F8 as the respective levels (i.e., the quadratic term assesses midline power relative to the average of left and right; while the linear term assesses left-right differences).

In previous work (Neo and McNaughton, 2011), right frontal theta activity was detected in the 0.5 s period immediately after the onset of the stimuli that cued the start of a 3-s countdown before the forced choice. Since changes across time were not examined, it was unclear if the observed theta was related to the previous trial or the upcoming choice. We therefore analyzed a factor, “Time” (**T**), which compared activity in the period of interest (0.5 s from the onset of the start cue) with the periods before and after. This comparison was reduced to a single df term using a quadratic contrast.

#### Stepwise Regressions

We extracted Cook’s distance and leverage values to determine potential outliers. Seven participants with leverage value three times over k + 1/n and Cook’s distance over 4/n were identified (k is the number of predictor variables and n is the number of observations). We used their behavioral responding as a basis for exclusion as it showed a significant relationship (see results). Six of the participants showed adaptive behavioral responding, adopting strategies normally seen in the tasks. A female participant was excluded as she showed an unusually low number of left-clicks across all the payoff conditions (average of 5 clicks in each condition).

## Results

### Behavioral Responses to the Payoff Conditions GAIN, CONFLICT, and LOSS

**FIGURE 2:**
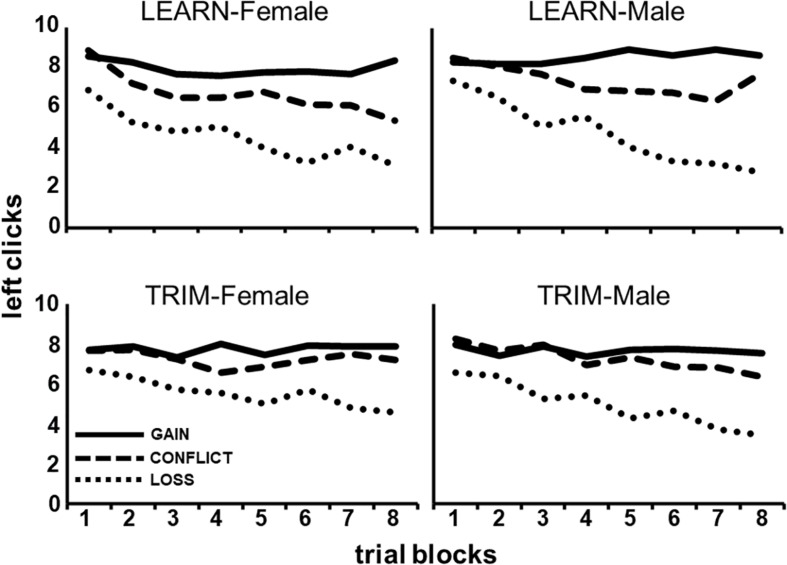
The average number of left clicks across eight, 10-trial, blocks for each of the three payoff conditions (GAIN, CONFLICT, and LOSS) for each gender and task (LEARN, TRIM).

As can be seen in Figure 2, males and females responded differently to payoff conditions over the trial-blocks, averaged across the Choice tasks [**P**(lin) × **B**(lin) × **G**, *F*(1,86) = 4.80, *p* < 0.05]. *Post hoc* tests show that males, compared to females, showed a steeper decrease in left clicking over trial-blocks in LOSS [**B**(lin) × **G:** GAIN, *F*(1,88) = 0.06, *p* = 0.80; CONFLICT, *F*(1,88) = 0.56, *p* = 0.46; LOSS, *F*(1,88) = 4.49, *p* < 0.05].

Figure 2 also shows that LEARN, compared to TRIM, showed a larger increasing separation between payoff conditions over trial-blocks, averaged across gender [**P**(lin) × **B**(lin) × **C**, *F*(1,86) = 3.80, *p* < 0.05]. *Post hoc* tests show that this was a result of a steeper decline in LOSS left clicking over trial-blocks in LEARN [**B**(lin) × **C:** GAIN, *F*(1,88) = 0.03, *p* = 0.86; CONFLICT, *F*(1,88) = 2.92, *p* = 0.09; LOSS, *F*(1,88) = 4.07, *p* < 0.05].

### EEG Effects Common Across Tasks and Gender

As mentioned before, we tested more participants with TRIM to address the unexpected large number of data lost due to EEG artifacts in the late task phase of LEARN. Task differences were therefore not a key focus of our study. Hence, we focus our report here only on effects that were common across tasks and gender that did not show higher order task-related interactions. Note also that the period of interest under study here was chosen for fair comparison across the tasks. For readers interested in interactions between gender and task, a summary of the full ANOVA statistics and supporting figures can be found in Supplementary Material.

**FIGURE 3:**
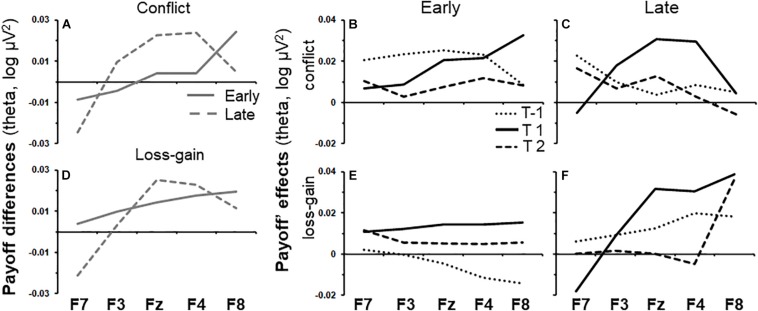
Variations of payoff effects across recording sites (F7, F3, Fz, F4, and F8) and time periods for early and late task phases. **(A)** Conflict theta activity specific to the mid time point, calculated as the average of T-1 and T2 subtracted from T1. **(B,C)** show variations of conflict theta activity for each time period of interest, for the early and late task phases, respectively. T1 is the 0.5 s period from the onset of the trial-start stimulus. T-1 and T2 indicate the 0.5 s periods before and after T1, respectively. **(D–F)** as **(A–C)** but for the loss-gain activity difference rather than for conflict activity.

Early task phase conflict activity and late task phase loss-gain activity were the only two activities that did not differ across tasks and gender, and both were in the theta band. As shown by the solid line in Figure 3A, the effect of time period on early phase conflict activity increased steadily across the recording sites from F7 to F8 [**T**(quad) × **P** × **S**(lin), *F*(1,86) = 6.73, *p* < 0.05]. The same trend (dotted line) was not observed in the late task phase. Details of the summarized effects in Figure 3A are shown in Figures 3B,C, which show the change in activity over the recording sites for each time period and task phase, respectively. The effect of time period on late phase loss-gain activity is indicated by the dotted line in Figure 3D. The increase of activity from F7 to F8 was reliable [**T**(quad) × **P** × **S**(lin), *F*(1,72) = 5.08, *p* < 0.05] and a similar trend was not detected in the early phase. As per above, the details of the summarized effects in Figure 3D are shown in Figures 3E,F.

We conducted *post hoc* tests to assess if conflict and loss-gain theta significantly change over the time periods, separately, for each individual recording site in the early and late task phases. The F-ratios are summarized in Table 1 below. Notably, only F8 early phase conflict theta showed a reliable time difference (see highlight in Table 1).

**TABLE 1:** Summary of *post hoc* tests. The values shown are F-ratios for the interaction between the quadratic contrast of time period (“Time”) and the respective payoff conditions (“Payoff”).

	**Conflict**		**Loss-gain**	
	**Early**	**Late**	**Early**	**Late**
F7	0.54	2.96	0.18	1.5
F3	0.03	0.02	0.31	0.2
Fz	0.72	0.36	0.03	0.68
F4	0.4	0.41	0.1	1.1
F8	6.8*	0.02	0.03	0.05

The degrees of freedom for analyses of early and late phase activity are early phase: 1, 89; and late phase: 1, 75. *p < 0.01.

### F8 Early Conflict Theta: Correlations With Behaviors and Personality Traits

**FIGURE 4:**
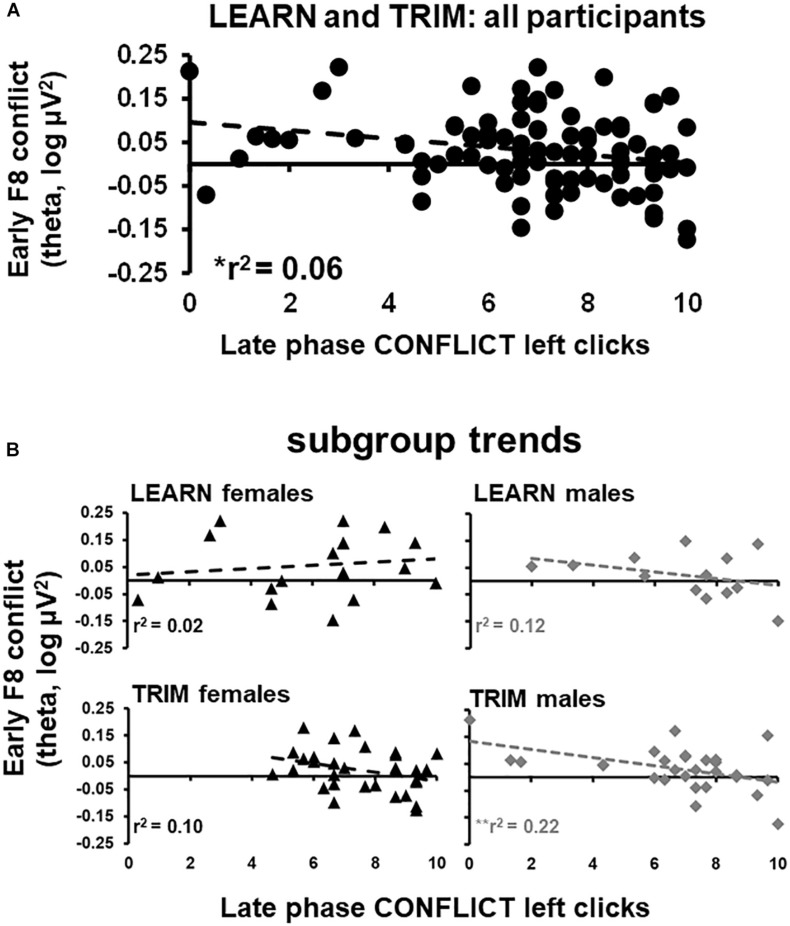
Scatterplots of early phase conflict theta activity and late phase left clicks in CONFLICT. **(A)** scatterplot for all participants in both LEARN and TRIM. **(B)** Scatterplots for each subgroup: LEARN females, LEARN males, TRIM females and TRIM males. Trend lines are indicated by dotted lines. *p < 0.05; **p < 0.01.

Early phase F8 conflict theta power, which showed a reliable peak in the period where the trial-start stimulus was presented, was submitted to a stepwise regression. Trait anxiety, neuroticism, early and late phase GAIN, CONFLICT, and LOSS left clicks were entered as predictor variables. F8 conflict power was negatively related to late phase left clicks in CONFLICT [*r*^2^ = 0.59, *F*(1,89) = 5.50, *p* < 0.05; see Figure 4A]. The stepwise regressions were repeated for females and males separately for LEARN and TRIM to determine if the relationship was driven by sub-groups [LEARN females: *r*^2^ = 0.02, *F*(1,16) = 0.34, *p* = 0.57; LEARN males: *r*^2^ = 0.12, *F*(1,11) = 1.44, *p* = 0.25; TRIM females: *r*^2^ = 0.10, *F*(1,28) = 3.19, *p* = 0.09; TRIM males: *r*^2^ = 0.22, *F*(1,26) = 7.51, *p* = 0.01]. As shown in Figure 4B, only females in LEARN showed opposite trends. People in the remaining groups all showed trends consistent with the main effect.

## Discussion

Here, despite the change from risk to ambiguity and the variation of probability rather than size of payoff, we replicated the general findings from the economic choice task of Neo and McNaughton (2011) and our unrewarded SST studies (Neo and McNaughton, 2011; Neo et al., 2011; McNaughton et al., 2013; Shadli et al., 2015b, 2016). That is, we observed GCSR in the right frontal region. Notably, the results were obtained in a large sample size, and did not differ across two *new* variants of the task used in Neo and McNaughton (2011), showing good generalization. The findings suggest that the right frontal region is involved in goal-conflict processing across domains. We also replicated specific findings from Neo and McNaughton (2011), detecting GCSR only in the early phase of training and in the theta band. GCSR observed in the early phase likely reflects active goal-conflict assessment or adaptation, which should be less dominant in the late phase once response strategy starts to stabilize (McNaughton, 1985).

More importantly, for the first time, right frontal GCSR showed a link with a neuroeconomic choice/decision bias. We think that the relationship observed here (and not in Neo and McNaughton, 2011), was a result of the increase in demand to search for information when decision-making has to be made under ambiguity (Huettel et al., 2006; Bach et al., 2009, 2011; Horga et al., 2011). Consistent with our theory of anxiety (Gray and McNaughton, 2000), people who showed more GCSR showed a preference for economic safety specific to the economic context of CONFLICT. This provides replicable, empirical evidence that a motivational system (BIS) other than pure loss (FFFS), can lead to negative, overt decision biases.

Contrary to our expectations, decision-making under ambiguity did not result in a relation of GCSR with either trait anxiety or neuroticism, casting doubt on whether the bias is anxiety-related. Anxiety can be measured in various ways (Polak et al., 2015; Heeren et al., 2018). If the right frontal GCSR observed here reflects processing specific to anxiety, our findings suggest it reflects an aspect of anxiety that is not effectively captured by the trait anxiety and neuroticism questionnaires. While we cannot rule out that the bias reflects other forms of emotional processing, this is unlikely since all the current forms of conflict processing being studied, such as goal-, response- and outcome- conflict processing (Gray and McNaughton, 2000; Cavanagh and Shackman, 2015), have been implicated in anxiety processes. These are not just different forms of conflict by definition, but also differ in terms of the regions that they have been commonly associated with (right frontal versus frontal midline). Consistent with a previous review of human frontal midline theta from the perspective of rat hippocampal theta (Mitchell et al., 2008), Beaton et al. (2018) found that in the flanker task, ventrolateral prefrontal but not frontal midline conflict theta activity, was sensitive to an anxiolytic, alcohol. It therefore appears that there are at least two and possibly more independent conflict mechanisms, and each of these could influence different aspects of anxiety processing.

Finally, our findings support the existing view of EEG theta as an electrophysiological mechanism for adaptive, cognitive control of flexible behavior, involved in conflict resolution. For example, in addition to right frontal GCSR, theta band activity, albeit from the frontal midline region, has been consistently observed during response- (Cohen, 2014), and outcome-conflict monitoring (Cavanagh and Frank, 2014). Theta band activity has also been observed in the lateral prefrontal regions in the same response- and outcome-conflict monitoring tasks (Nigbur et al., 2012; Tang et al., 2013; Beaton et al., 2018). However, conflict processing is not limited to theta processes. GCSR spanned into the alpha range during action inhibition in the SST. Conflict adaptation in the classical Stroop task also recruits higher frequencies (Tang et al., 2013) with right frontal theta activity in the “look” condition (no response required) and, additionally, right frontal alpha in the “do” condition (response required). Taken together with the observation of only theta band activity here, where goal-conflict was generated by slower decision-making processes, it appears that alpha frequencies are also recruited when the conflict is generated by faster motor processes (the SST involves speeded responses). However, it is unclear if the shift in frequencies is a result of the motor processes *per se* or a result of physiological arousal generated by time-pressured actions.

To conclude, the BIS is neurally detailed. However, how it impacts economic decisions is not well understood; and remains unexplored within decision neuroscience. Here, we provide the first demonstration that goal-conflict theta activity is linked to a decision bias. Both the brain and behavioral measures used were distinct from gain and loss, *per se*. The findings here are a crucial demonstration of BIS applicability and integration with neuroeconomics. They provide a new lens through which to view decision biases, and should help us to dissect choice processes, and the associated emotional processing more precisely. Conversely, we did not find direct evidence that the conflict-decision bias link here is an anxiety process. However, taken together with previous studies, goal-conflict appears to be one of multiple independent conflict mechanisms, which share common electrophysiological features, such as the recruitment of EEG theta activity for adaptive cognitive control. Notably, all of the conflict mechanisms have previously been linked to anxiety processing. It is likely that the bias observed here is linked to anxiety, albeit, one that is not effectively captured by the self-report measures of trait anxiety and neuroticism used here.

## Data Availability Statement

The raw data supporting the conclusions of this article will be made available by the authors, without undue reservation, to any qualified researcher.

## Ethics Statement

These studies were reviewed and approved by the Lower South Regional Committee (LRS/09/05/017) and the University of Otago Human Ethics Committee (OTA/04/03/019). The participants provided their written informed consent to participate in this study.

## Author Contributions

PN collected, processed, and analyzed the data and prepared the manuscript. JT collected and processed the data. NM provided academic supervision and guidance for the experimental design, data collection, analyses and preparation of the manuscript, and edited the manuscript.

## Conflict of Interest

The authors declare that the research was conducted in the absence of any commercial or financial relationships that could be construed as a potential conflict of interest.
