# Supplementary material for: Goal-Conflict EEG Theta and Biased Economic Decisions: A Role for a Second Negative Motivation System
Source: Front Neurosci. 2020 Apr 15;14:342. doi: 10.3389/fnins.2020.00342 (PMC7174654; doi:10.3389/fnins.2020.00342)
Supplement: Supplementary file 1 [file Data_Sheet_1.PDF]

Supplementary materials

1 Supplementary Figures and Tables

Table S1. Summary of ANOVA statistics.

	Conflict activity				Loss-gain activity			
	Early		Late		Early		Late	
Effects	Theta	Alpha	Theta	Alpha	Theta	Alpha	Theta	Alpha
T(quad) × P	0.37	2.91	0.74	1.08	0.00	3.13	0.05	0.01
T(quad) × P × G	0.60	0.01	0.57	0.46	2.37	2.80	2.98	0.03
T(quad) × P × C	0.52	2.35	5.17 *	4.81 *	0.44	2.74	0.48	0.03
T(quad) × P × G × C	0.21	0.00	0.07	0.78	0.09	4.15 *	0.62	0.10
T(quad) × P × S(lin)	6.73 *	0.13	2.34	0.11	1.91	0.66	5.08 *	0.04
T(quad) × P × S(quad)	0.43	1.66	7.44 **	0.56	0.03	0.08	2.45	0.18
T(quad) × P × G × S(lin)	0.39	4.76 *	6.41 *	0.07	2.55	0.93	1.40	3.53
T(quad) × P × G × S(quad)	0.28	2.65	1.51	0.95	4.69 *	0.68	1.04	0.01
T(quad) × P × C × S (lin)	0.82	0.24	0.02	0.68	6.95 *	0.41	2.25	1.63
T(quad) × P × C × S(quad)	0.09	0.09	9.58 **	0.05	0.35	0.72	1.30	0.28
T(quad) × P × G × C × S(lin)	3.15	3.03	7.14 *	0.78	0.04	0.73	1.01	1.93
T(quad) × P × G × C × S(quad)	0.00	0.18	2.46	4.31 *	0.04	0.20	0.40	0.02

The values shown are F-ratios. The degrees of freedom for analyses of early and late phase activity are early phase: 1, 86; late phase: 1, 72. P, 'Payoff'; G, 'Gender'; S, 'Site'; T, 'Time', C, 'Choice task'. *p < 0.05; **p < 0.005 (not Bonferroni corrected; early theta conflict activity was a predicted effect). Effects common across tasks and gender are highlighted.

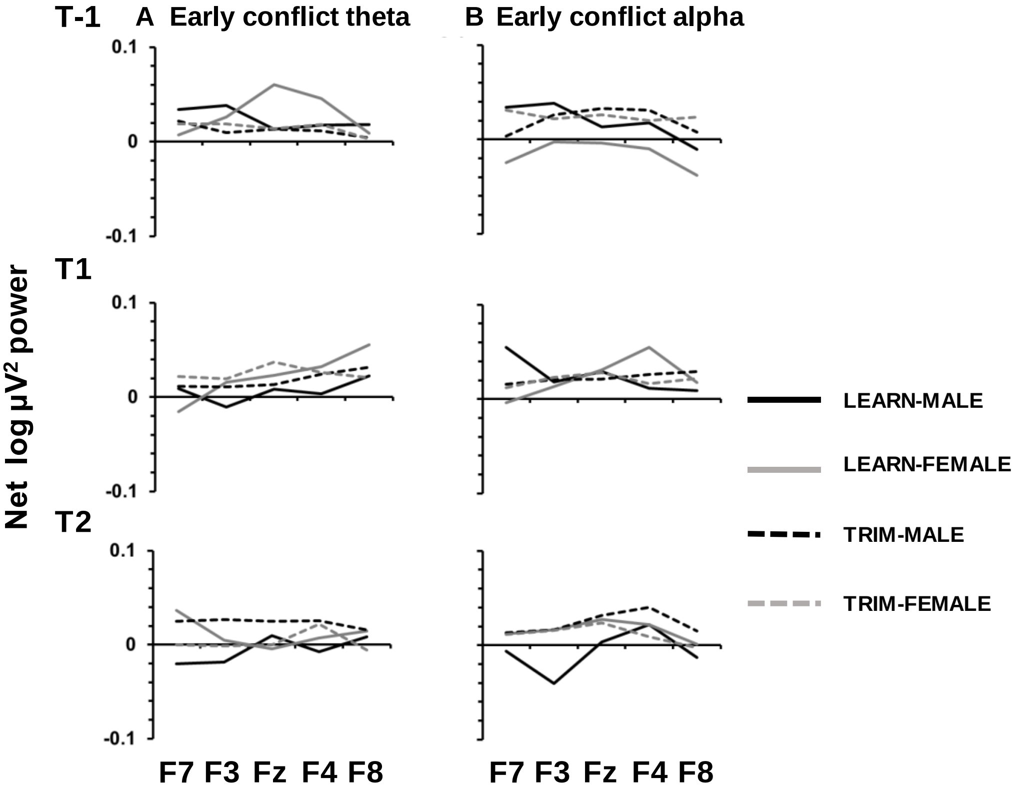

Figure S1. Variations of goal-conflict activity across the frontal scalp regions in the early phase. T1: 0.5s from the onset of the start-trial stimuli. T-1 and T2 indicate the 0.5s period immediately before and after T1.

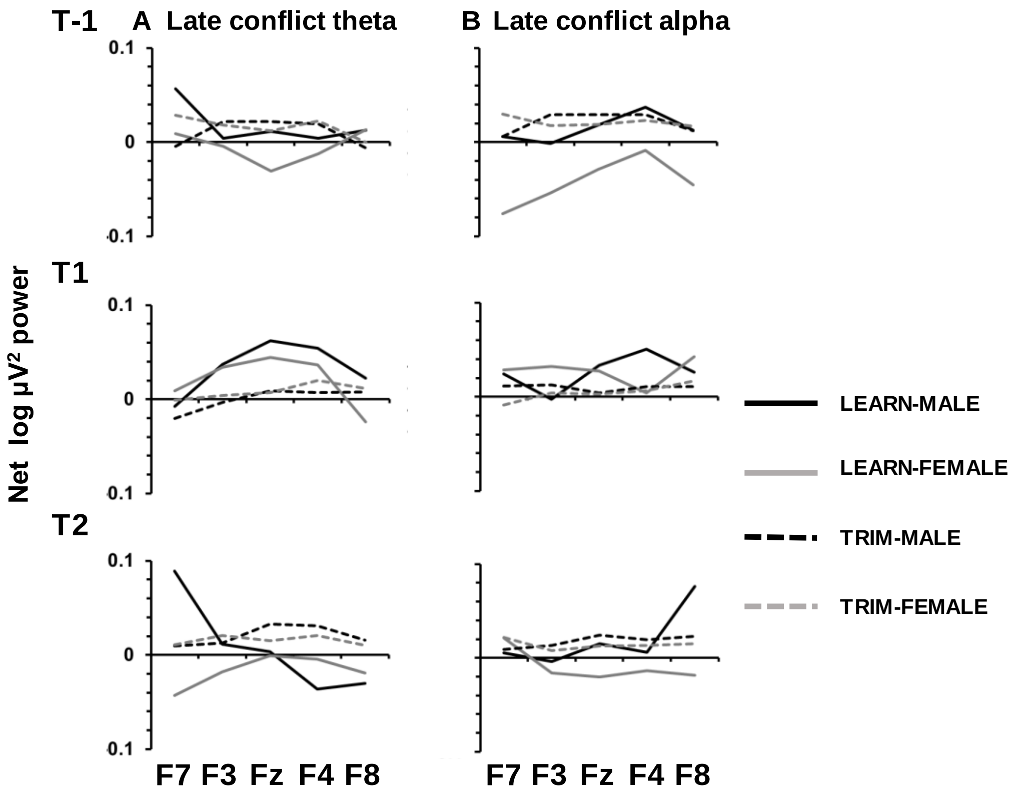

Figure S2. Variations of goal-conflict activity across the frontal scalp regions in the late phase. T1: 0.5s from the onset of the start-trial stimuli. T-1 and T2 indicate the 0.5s period immediately before and after T1.

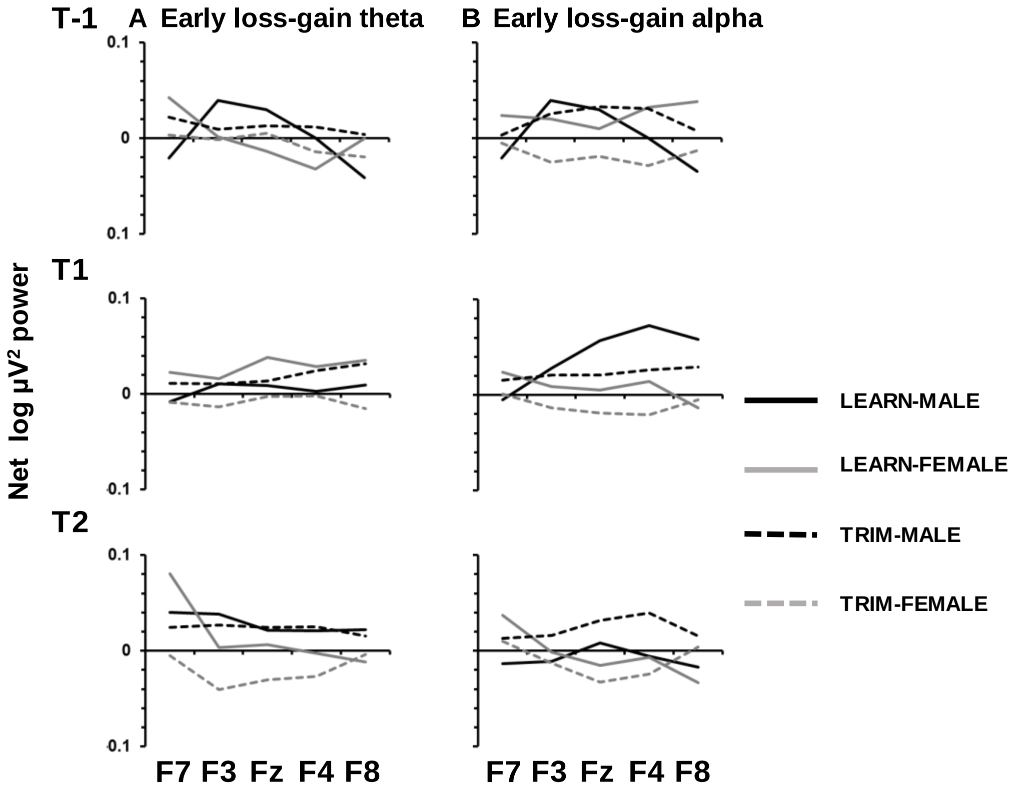

Figure S3. Variations of loss-gain activity across the frontal scalp regions in the early phase. T1: 0.5s from the onset of the start-trial stimuli. T-1 and T2 indicate the 0.5s period immediately before and after T1.

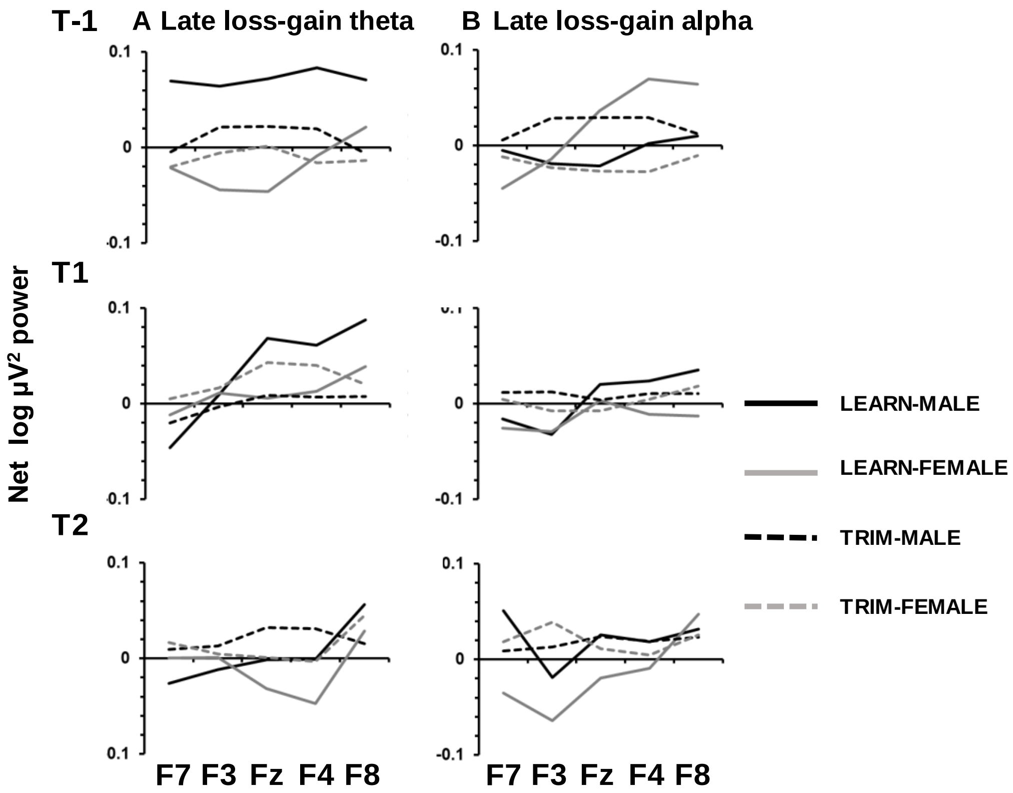

Figure S4. Variations of loss-gain activity across the frontal scalp regions in the late phase. T1: 0.5s from the onset of the start-trial stimuli. T-1 and T2 indicate the 0.5s period immediately before and after T1.
